# Barriers to and facilitators of HIV serostatus disclosure to sexual partners among postpartum women living with HIV in South Africa

**DOI:** 10.1186/s12889-021-10955-x

**Published:** 2021-05-13

**Authors:** Oladele Vincent Adeniyi, Charlotte Nwogwugwu, Anthony Idowu Ajayi, John Lambert

**Affiliations:** 1 Department of Family Medicine & Rural Health, Faculty of Health Sciences, Walter Sisulu University, Mthatha/East London Hospital Complex, Cecilia Makiwane Hospital, East London, South Africa; 2 Masters in Public Health Program, UMB School of Medicine, University of Maryland School of Nursing, 655 West Lombard St., Room 482, Baltimore, MD 21201 USA; 3 Population Dynamics and Sexual and Reproductive Health, African Population and Health Research Centre, APHRC Campus, Manga Close, Nairobi, Kenya; 4 Mater Misericordiae University Hospital and University College Dublin School of Medicine, Dublin, Ireland

**Keywords:** Disclosure, Open communication, Relationship status, HIV status, South Africa

## Abstract

**Background:**

Disclosure of HIV serostatus to a sexual partner can facilitate partner’s support and testing and better treatment outcomes. Studies examining changes in disclosure rates of serostatus from delivery and postpartum periods are scarce. Our study fills this gap by using a follow-up survey of postpartum women with HIV to examine if disclosure prevalence has improved compared to the proportion recorded at childbirth. We further assessed the reasons for non-disclosure and correlates of serostatus disclosure to sexual partners.

**Methods:**

We conducted a cross-sectional analytical study (exit interview) with a final sample of 485 postpartum women with HIV drawn from the East London Prospective Cohort study database between January and May 2018. Disclosure of HIV status to partner was based on self-reporting. We fitted adjusted and unadjusted logistic regression models and also conducted descriptive statistical analyses. Sampling weights were used to correct for sampling errors.

**Results:**

Overall, 81.8% of women in the study cohort had disclosed their status to their partners, representing a 7.4 percentage point increase since child delivery. After adjusting for important covariates, women were more likely to disclose their status if they were married [adjusted odds ratio (AOR): 3.10; 95% confidence interval (CI):1.39–6.91] but were less likely to disclose if they used alcohol [AOR: 0.61; 95% CI:0.37–0.99] or had reported adherence to ART [AOR: 0.59; 95% CI:0.36–0.96]. Fear of rejection, stigma or being judged, new or casual relationships, and having a violent partner were the main reasons for not disclosing HIV status to sexual partners.

**Conclusion:**

We found a relatively higher rate of HIV status disclosure in the cohort compared to the rate recorded at childbirth, suggesting an improvement over time. Also, complicated relationship dynamics and fear of social exclusion still constitute barriers to HIV status disclosure to sexual partners despite patients’ counselling.

**Supplementary Information:**

The online version contains supplementary material available at 10.1186/s12889-021-10955-x.

## Background

Disclosure of HIV serostatus has substantial implications for health outcomes, particularly in reaching the goal of an AIDS-free generation. Evidence shows that disclosure of HIV status promotes voluntary testing, safer sexual practices, and improves adherence to antiretroviral therapy (ART) [1]. HIV status disclosure has also been linked to positive mental health outcomes as social support from family and social networks has been shown to improve psychological well-being [2]. However, despite the documented benefits of serostatus disclosure, rates of disclosure to partners vary widely in sub-Saharan Africa. Among pregnant and postpartum African women living with HIV, disclosure rates to any individual range between 5 and 97%, and to male partners, rates range between 30 and 93% [3].

While it is critical that people living with HIV disclosed their status to their partner, stigma—both real and perceived— and social exclusion of people with HIV may hinder disclosure of status [4–6]. Fear of abandonment and sex deprivation, emotional abuse, intimate partner violence [7–9] results in non-disclosure of HIV status to their partners. Non-disclosure is usually dependent on the woman’s previous experience in terms of the direct observation of the maltreatment of others, which includes, but is not limited to, social ostracisation and gossiping [5, 6, 10].

The Joint United Nations Programme on HIV/AIDS (UNAIDS) had set a target of having 95% of people living with HIV knowing their serostatus, 95% of people who know their status to receive treatment and 95% of people on HIV treatment have a suppressed viral load [11]. These are not lofty goals, but one achievable with political commitment and inclusive society that does not stigmatize persons living with HIV. Supporting postpartum women to disclose their status will in no small measure contribute to achieving these goals.

The postpartum period is crucial for implementing interventions targeting vertical HIV transmission through breastfeeding and horizontal transmission. Breastmilk transmission of HIV remains a major threat during the immediate postpartum period; thus, serostatus disclosure to partners will further enhance adherence support and reduce the risk of vertical transmission [2]. Besides, serostatus disclosure to partners would improve the adoption of safe sexual practices and partner testing, which are important strategies in preventing horizontal transmission at the community level [2, 12]. Couple testing was adopted as one of several strategies for the prevention of mother-to-child transmission (PMTCT) in South Africa [13]; however, men often present late for HIV testing compared to women in the country [14]. Also, a high proportion of women (between 15 and 49 years) in South Africa are single (64%) and may not be in committed or long-term relationships [15, 16]. Therefore, strengthening of serostatus disclosure to partners through counselling and education of women would further consolidate the goal of PMTCT.

In South Africa, further counselling on HIV disclosure is offered after delivery to strengthen ART adherence and prevent HIV transmission as per the PMTCT guidelines [13]. While several studies have investigated the prevalence and correlate of serostatus disclosure in South Africa [1, 3, 15, 17–20], we did not find a study that followed up a cohort of women to track improvement in the rate of disclosure to partners over a period of time. We conducted a follow-up (exit interview) study of the women enrolled in the East London Prospective Cohort Study in the Eastern Cape, South Africa, to fill this gap. We also examined reasons for non-disclosure and correlates of status disclosure to a sexual partner. These findings might shed light on the context in which disclosure decisions occur in South Africa to develop interventions that support women in making decisions about HIV disclosure during a vulnerable period of their lives. These findings could also highlight new avenues to tackle the barriers mitigating against HIV serostatus disclosure for the broader population of people living with HIV.

## Methods

### Study design and settings

This cross-sectional analytical study (follow-up exit interview) was conducted between January to May 2018 on a sample of parturient women with HIV enrolled in the electronic database of the East London Prospective Cohort Study [21]. All pregnant women attending maternity service would have received HIV testing, commenced on ART if diagnosed with HIV, and documented in the antenatal medical records per the PMTCT guidelines [13]. An electronic database was created for research purposes by the investigators between September 2015 and May 2016 to track the PMTCT outcomes of parturient women with HIV and their infants in three hospitals in Buffalo City and the Amathole district of the East Cape Province of South Africa. These hospitals serve a combined population of 1.7 million people residing in the rural and urban communities of the central region of the Eastern Cape [22].

### Participants and sample size

The sample size for this sub-study (exit interview) was estimated as 485, using the Cochran formula for categorical data, at a confidence level of 95%, a precision level of +/− 4 and 10% possible attrition. All the women enrolled in the East London Prospective Cohort Study database (*N* = 1709) signed informed consent at the baseline to be contacted for the follow-up (exit) study. However, only those who were accessible telephonically were considered eligible for this follow-up survey. Each participant was offered a choice to either complete an interviewer-guided interview face-to-face or telephonically. A few participants (*n* = 43) who chose to attend interviews at one of the three hospitals were reimbursed for transportation costs. Those who chose to complete telephonic interviews agreed to a scheduled time with our research team. A convenience sampling of the participants who were available and willing to participate was conducted.

We employed and trained two research assistants, who were fluent in both IsiXhosa (local language) and English for this study. The research team successfully contacted 509 participants out of a total of 1709 participants (29.9%). Some of the eligible participants were no longer accessible through any of the three contactable mobile numbers obtained from the electronic database. We designed a questionnaire specifically for the exit interview, which was piloted with 12 parturient women with HIV in one of the hospitals to ascertain the validity and reliability of the instrument. We subsequently adjusted the questionnaire using feedback from the participants and the investigators.

### Measures

With the aid of a validated questionnaire (Supplementary 1), outcome (dependent) and independent variables were obtained from the interview. These variables were informed by the existing knowledge in the literature and are described briefly.

#### Outcome variable

The key outcome variable for this study was disclosure of HIV to a sexual partner, and this was measured by asking the participants if they had disclosed their serostatus to their sexual partners. Responses were categorized as “yes” or “no” which was coded as 1 for ‘yes’ and 2 for ‘no’. Also, we used an open-ended question to elicit information on the reasons for non-disclosure.

#### Independent variables

Socio-demographic, lifestyle behaviours and clinical characteristics were the covariates included in this study. The selection of these covariates was based on the existing knowledge of HIV disclosure in the literature [8–11].

#### Socio-demographic characteristics

We obtained information on the participants’ ages which were coded as continuous variables, level of education, and marital status. We obtained information on the employment status of the participants, occupation in the preceding 12 months at the time of the study, and whether they were engaged in a salary-paying job. We obtained additional information on whether participants were receiving child support grants (social grants) from the South African government.

#### Lifestyle behaviours

We obtained information on the smoking status and alcohol consumption as categorical data with “yes” or “no” response, which was coded as 1 for ‘yes’ and 2 for ‘no’. Smoking status was obtained by self-report of cigarette or any tobacco product within the preceding month of the study. Similarly, the consumption of any alcoholic beverage in the prior month of the study was obtained by self-report and recorded.

#### Clinical characteristics

The following clinical information was obtained through self-reporting by the participants: awareness of partner’s HIV serostatus and complete adherence to ART (no missed dose of ART in the preceding week of the study). A binary response of “yes” or “no” was provided for the participants, which was coded as 1 for ‘yes’ and 2 for ‘no’. We also documented the duration of HIV infection (period since diagnosis) among the participants, which was categorized as a continuous variable. We asked participants to provide open responses to reasons for not disclosing their status to their respective partners.

### Data analysis

Data analysis was performed on responses from 485 out of 509 participants contacted (95.3%). Of the 24 participants excluded in the analysis, nine were not interested in discussing their HIV serostatus and thus, withdrew during the interview. Six participants refused to participate in the exit interview, and nine were confirmed to have died by the family member who responded to the phone call. Disclosure of HIV serostatus to sexual partners was the main outcome measure of this study. Complete responses were available for 485 respondents on the main outcome measure and were included in this analysis. All analyses were conducted with the IBM Statistical Products and Service Solutions, version 27.0 (SPSS, Chicago, IL, USA). Given that this data is a convenient sample of the 1709 women with HIV who gave birth between September 2015 and May 2016 in the three largest health facilities in Eastern Cape, South Africa, we calculated sampling weight using respondents’ age distributions. We applied the sampling weight for all analyses.

Descriptive statistics (means, frequency, and percentages) were used to summarise the characteristics of the participants disaggregated by their disclosure status. Given that the main outcome variable is dichotomous (“yes or no”) and there are many explanatory variables, we performed adjusted and unadjusted logistic regression models to determine the independent and significant influencing factors of non-disclosure in the study. Variables were included in the final model if they have been shown by previous studies to influence disclosure of status significantly. Knowing partner status was positively correlated with the outcome variable and was therefore removed from the multivariate analysis. Also, smoking behaviour was highly related to alcohol use and was removed from the model. The 95% confidence intervals were reported for all analyses, and *p*-values less than 0.05 were considered statistically significant.

## Results

### Socio-demographic characteristics

The average age of participants was 30.07 ± 5.85 years. Most participants were single (78.9%), had up to grade 12 levels of education (88.6%), were unemployed (71.3%), but received social grants (92.9%). The majority of the participants knew their partner’s HIV status (65.2%), did not smoke cigarettes in the past month (90.6%), had consumed alcoholic beverages in the past month (63.2%), had been living with HIV for 1 to 5 years (53.2%) and self-reported complete adherence to ART (58.6%) (Table 1).

**Table 1:** Demographic and clinical characteristics of study participants

Variables	Unweighted frequency	Unweighted proportions	Weighted frequencies	Weighted proportions
All	485	100	486	100
Age				
24 years and less	36	7.4	112	23.0
25–29 years	114	23.5	131	27.0
30–34 years	144	29.7	130	26.7
35–39 years	123	25.4	86	17.7
40 years and above	68	14.0	27	5.6
Marital status				
Single	359	74.0	384	78.9
Married	126	26.0	102	21.1
Education level				
Grade 7 and less	30	6.2	21	4.3
Grade 8–12	421	86.8	431	88.6
Higher education	34	7.0	34	7.1
Employed in a salary paying job				
Yes	157	32.4	139	28.7
No	328	67.6	347	71.3
Occupation in last 12 months				
Government employee	17	3.5	14	2.8
Non-government employee	114	23.5	103	21.2
Self-employed	29	6.0	26	5.4
Student	23	4.7	44	9.1
Unemployed	302	62.3	299	61.5
Receives government social grant				
Yes	453	93.6	451	92.9
No	31	6.4	35	7.1
Smoked in the past month				
Yes	43	8.9	46	9.4
No	442	91.1	440	90.6
Drank alcohol in the past month				
Yes	173	35.7	307	63.2
No	312	64.3	179	36.8
Knows partner’s serostatus				
Yes	319	65.8	317	65.2
No	166	34.2	169	34.8
Year since HIV diagnosis				
1–5 years	206	42.5	259	53.2
6–10 years	163	33.6	145	29.8
11–17 years	116	23.9	83	17.0
Complete adherence				
Yes	310	63.9	285	58.6
No	175	36.1	201	41.4

*Source*: Exit interview of the East London Prospective Cohort Study done (2018)

### Level of serostatus disclosure to partners

A total of 397 participants (81.8%) had disclosed their HIV serostatus to their partners. However, the proportion of respondents who had disclosed their status varies by age, marital status, alcohol use, and knowing a partner’s status. The HIV disclosure rate was highest among women who were married (92.2%), had higher education (88.2%), knew their partner’s status (97.5%), and self-reported complete ART adherence (86.0%) (Table 2).

**Table 2:** Weighted Pearson Chi-square analysis of factors associated with HIV status disclosure among postpartum women with HIV

Variables	Disclosed serostatus to partner	Had not disclosed serostatus to partner	***p***-value
All	397 (81.8)	89 (18.2)	
Age			
24 years and less	84 (75.0)	28 (25.0)	0.182
25–29 years	113 (86.3)	18 (13.7)	
30–34 years	110 (84.6)	20 (15.4)	
35–39 years	69 (80.2)	17 (19.8)	
40 years and above	22 (78.6)	6 (21.4)	
Marital status			
Single	303 (78.9)	81 (21.1)	0.001
Married	95 (92.2)	8 (7.8)	
Education level			
Grade 7 and less	18 (85.7)	3 (14.3)	0.531
Grade 8–12	350 (81.2)	81 (18.8)	
Higher education	30 (88.2)	4 (11.8)	
Employed in a salary paying job			
Yes	107 (77.0)	32 (23.0)	0.053
No	290 (83.8)	56 (16.2)	
Occupation in last 12 months			
Government employee	9 (64.3)	5 (35.7)	0.0116
Non-government employee	82 (79.6)	21 (20.4)	
Self-employed	22 (84.6)	4 (15.4)	
Student	32 (72.7)	12 (27.3)	
Unemployed	253 (84.6)	46 (15.4)	
Receives government social grant			
Yes	366 (81.0)	86 (19.0)	0.099
No	31 (91.2)	3 (8.8)	
Smoking in the past month			
Yes	33 (73.3)	12 (26.7)	0.119
No	364 (82.7)	76 (17.3)	
Drank alcohol in the past month			
Yes	134 (75.3)	44 (24.7)	0.003
No	263 (85.7)	44 (14.3)	
Knows partner’s serostatus			
Yes	309 (97.5)	8 (2.5)	< 0.001
No	88 (52.1)	81 (47.9)	
Year since HIV diagnosis			
1–5 years	208 (80.3)	51 (19.7)	0.415
6–10 years	118 (81.4)	27 (18.6)	
11–17 years	72 (86.7)	11 (13.3)	
Complete adherence			
Yes	245 (86.0)	40 (14.0)	0.004
No	153 (76.1)	48 (23.9)	

*Source*: Exit interview of the East London Prospective Cohort Study done (2018)

### Factors influencing HIV serostatus disclosure

We used adjusted and unadjusted logistic regression models to examine the factors influencing HIV serostatus disclosure to a sexual partner. We included demographic, behavioural, and clinical factors in the model based on previous studies indicating that these factors were associated with disclosure of HIV status [1, 3, 15, 17–20]. However, we removed knowing partner’s HIV status from the model due to colinearity with the disclosure of HIV status. Also, we removed smoking due to colinearity with alcohol use. In the adjusted regression, being married [unadjusted odds ratio (UOR): 3.29, 95% confidence interval (CI) 1.51–7.13] was associated with increased odds of HIV serostatus disclosure to partner, while alcohol use (COR: 0.51, 95% CI 0.32–0.81) and adherence to ART (UOR: 0.52, 95% CI: 0.33–0.83) were associated with reduced odds of HIV serostatus disclosure (Table 3). The magnitude and direction of the effects of these variables remain in the adjusted model. Married women were three times more likely to disclose their status to their partners relative to single women. However, alcohol users were 39% less likely to disclose their status compared to non-users of alcohol. Women who reported adherence to ART were 41% less likely to disclose their status.

**Table 3:** Adjusted and unadjusted logistic regression models showing the correlates of HIV serostatus disclosure to sexual partner

Variables	Unadjusted Odds ratios	Adjusted Odds ratios
Marital status		
Married	3.29 (1.51–7.13)*	3.10 (1.39–6.91)*
Single	1	1
Age		
18–34 years	1.14 (0.67–1.95)	1.66 (0.89–3.11)
35–46 years	1	1
Education level		
Grade 7 and less	0.89 (0.18–4.34)	0.52 (0.10–2.78)
Grade 8–12	0.63 (0.23–1.77)	0.58 (0.20–1.67)
Higher education	1	1
Employed in a salary paying job		
Yes	0.64 (0.40–1.05)	0.67 (0.40–1.12)
No	1	1
Drank alcohol in the past year		
Yes	0.51 (0.32–0.81)*	0.61 (0.37–0.99)*
No	1	
Year since HIV diagnosis		
1–5 years	0.60 (0.29–1.22)	0.62 (0.28–1.40)
6–10 years	0.65 (0.30–1.39)	0.65 (0.29–1.48)
11–17 years	1	1
Complete adherence		
Yes	0.52 (0.33–0.83)*	0.59 (0.36–0.96)*
No	1	1

*Source*: Exit interview of the East London Prospective Cohort Study done (2018)

*p-value < 0.05

### Reasons for non-disclosure

The main reasons for not disclosing HIV serostatus to sexual partners were: lack of readiness, fear of rejection and violent reaction from the partner, fear of breaking up the relationship and being in a casual relationship (Table 4). Women who had not disclosed their status were seriously concerned about how their partner will react if they were to learn of their status, fearing rejection and violence against them. As a result, non-disclosure is seen as beneficial, enabling them to maintain peace and protect their relationships. Their fear of partner violence is not only perceived but real, as a few participants had observed their partner say unpleasant things or expressed judgemental attitudes about people living with HIV.

**Table 4:** Reasons for non-disclosure of HIV serostatus

Reasons for not disclosing serostatus	Frequency ***n*** = 89	Percentage
Fear of rejection and violent reaction	14	16.5
Broke up with him	10	11.8
Not ready to tell him	55	64.7
Not that close to him to discuss such topic	6	7.8

*Source*: Exit interview of the East London Prospective Cohort Study done (2018)

When asked why she had not disclosed her status, one participant responded: “*I am afraid to tell because he says nasty things about people with HIV*”. Also, a few others were experiencing emotional abuse in their relationship at the time of the study, heightening their fear of possible violence if they disclosed their status. In contrast, some were in new relationships and did not consider themselves to be close enough to share such private information with their partners. While others simply did not know how to broach such a topic with their partners because they just do not talk about such a subject. Lastly, some women believed that they do not need to disclose their status because they were in relationships with no prospects and revealing such information was unnecessary.

## Discussion

Given the dynamic nature of HIV serostatus disclosure to significant others, especially a sexual partner, it is crucial to examine the extent to which disclosure had improved over a period of time and its influencing factors among the women enrolled in the East London Prospective Cohort Study in the Eastern Cape, South Africa. To our knowledge, this is the first study from the Eastern Cape province, South Africa, to follow up a cohort of postpartum women with HIV. Therefore, this study provides new insights into the contextual factors that influence serostatus disclosure to sexual partners in the region, which might guide interventions for the broader population of people living with HIV.

This study reports a serostatus disclosure rate of 81.8%. Given that HIV disclosure is an independent predictor of ART adherence, which mediates viral suppression [12, 20, 23], the disclosure rate reported in this study is commendable. Status disclosure to partners should be strengthened to prevent further transmission during the postpartum period (HIV breastmilk transmission) in the region by consolidating the counselling sessions at the postnatal clinics. As previously reported, non-disclosure is strongly associated with high viral load whilst on ART [12, 20, 23] and increased risk of HIV transmission to infants through the breastmilk. The high rate of disclosure in the present study (81.8%) is higher than the rate (74.4%) reported in the same population during the antenatal period by Adeniyi et al. [15]. Similar disclosure rates were reported in the literature among pregnant and postpartum women in France, Tanzania and Pretoria, South Africa [17, 18, 24]. Given that HIV status disclosure is a dynamic process that evolves over a period of time [2, 25], the present study reports a seven-point increase in the disclosure rate observed after 24 months post-delivery. This further demonstrates that individuals living with HIV become more open and comfortable with their partners, thus, facilitating discussion about their status [18, 26].

Our results show that being married was associated with increased odds of HIV status disclosure. Participants who had not disclosed their status indicated that they had broken up the relationship with their partner or were not close enough to warrant disclosure of HIV status. This finding is similar to previous studies conducted in Tanzania, Ethiopia, Kenya, and Burkina Faso [7, 10, 19, 27]. Daily contact in marital relationships might increase the likelihood of HIV status disclosure compared to single women who may not necessarily be committed to the relationship. The dynamics in the relationship status of women with HIV in the region are unique. The majority were single (74%) and as reported in the qualitative component; some of these women did not envisage a future with the current partner, some consider their relationships as casual and others felt that they had not attained the level of closeness to broach the topic of HIV status with their partner. It is, therefore, imperative for clinicians and programme managers to provide the necessary support for single women with HIV. Given that a large proportion (64%) of women between 15 and 49 years are single in South Africa [16], context-specific interventions should be crafted to promote serostatus disclosure in this vulnerable population in the region.

Surprisingly, the duration of the infection did not influence the disclosure rate observed in this study, as the majority of the participants (57.5%) had been diagnosed more than 5 years at the time of this survey. Nevertheless, all participants had been diagnosed with HIV for over 2 years, suggesting that they had had sufficient time to process their HIV status and decide on who and when to disclose their status. Perhaps, the length of the relationship with a current partner might shed light on the association between infection duration and disclosure of HIV status. Whether the change of sexual partners after the diagnosis mediated the association between HIV status’ disclosure and duration of infection is unclear in this study.

Behavioural challenges such as alcohol use and adherence to ART were associated with non-disclosure in this study. Findings on the influence of alcohol on non-disclosure of status are mixed, with some studies reporting no significant association [28–30] and others reporting positive association [15]. Regardless of the previous reports, any individual who does not disclose his/her serostatus and continues to practice unprotected sex [31], which is very common among alcohol users, is highly likely to transmit HIV. To prevent new infections at the community level; whether vertical (in the postpartum period) or horizontal transmission, disclosure to sexual partners must be encouraged and prioritized by public health officials. The relationship between ART adherence and disclosure of HIV serostatus is well established. Hence, this finding supports previous reports on disclosure and ART adherence [12, 20, 23]. The PMTCT guidelines should therefore focus on encouraging women to disclose their status with a view of promoting couple testing [13].

Reasons for non-disclosure among the participants were fear of rejection and partners’ violent reactions and not being ready to disclose. These are consistent with previous studies in a similar setting in South Africa [15] and elsewhere [17, 32]. Addressing these reasons through counselling might help facilitate the disclosure of HIV status to partners. The ultimate goal of status disclosure is to motivate partners to get tested for HIV and access treatment if tested positive, which is in line with the UNAIDS 95–95-95 goal of achieving an HIV-free generation. Also, achieving undetectable viral load levels has been proven to eliminate the chances of HIV transmission with the Undetectable = Untransmittable campaign [33]. Educating the public on this important advancement in HIV treatment will help to reduce stigma and facilitate HIV disclosure and testing [33].

### Study limitations

The cross-sectional nature of the data limits the determination of the temporal nature of the associations reported in this study. Also, a large proportion of the women were no longer accessible, which may impact the representativeness of the participants in this study. The outcome variable was measured by self-reporting; as such, social desirability and recall bias cannot be excluded entirely, though this is a common phenomenon. Nevertheless, this study provides essential insights into the influencing factors of serostatus disclosure by the broader population of women living with HIV and other populations in the region. Findings will guide health authorities to develop strategies to provide HIV prevention programmes in this population and the broader population of people living with HIV in the region.

## Conclusions

We found a relatively higher rate of HIV status disclosure in the study population compared to the rate recorded at childbirth suggesting that individuals living with HIV become more open and comfortable with their partners over time, thus, facilitating discussion about their status. Also, complicated relationship dynamics still present barriers to HIV status disclosure to sexual partners in this region. Clinicians’ counselling and education should focus on strengthening open communication between partners to improve status disclosure, HIV treatment outcomes and further prevent vertical transmission in this region. Intervention strategies should be tailored for single women with HIV to promote HIV disclosure, improve ART adherence, and, consequently, decrease transmission at the community level.

## Supplementary Information

**Additional file 1.** PMTCT Questionnaire (Adeniyi, et al., 2018).

## Data Availability

All data and materials used for this study are available with the corresponding author upon reasonable request.

## Abbreviations

AIDS: Acquired immune deficiency syndrome
ART: Anti-retroviral therapy
HIV: Human immunedefimmunodeficiency
MTCT: Prevention of mother-to-child transmission
SSA: Sub-Saharan Africa
