# Supplementary material for: Barriers to and facilitators of HIV serostatus disclosure to sexual partners among postpartum women living with HIV in South Africa
Source: BMC Public Health. 2021 May 13;21:915. doi: 10.1186/s12889-021-10955-x (PMC8117658; doi:10.1186/s12889-021-10955-x)
Supplement: Supplementary file 1 — Additional file 1. PMTCT Questionnaire (Adeniyi, et al., 2018). [file 12889_2021_10955_MOESM1_ESM.docx]

PMTCT Questionnaire (Adeniyi, et al., 2018)

| Name of Data Capturer | |  | | | Hospital Name |  | |
| --- | --- | --- | --- | --- | --- | --- | --- |
| Hospital No |  | | Form ID |  | | Age |  |

| **Q1** | **Marital Status:** | Single | |  | | Married |  | Cohabiting |  | | | | Previously married | | |  | | |
| --- | --- | --- | --- | --- | --- | --- | --- | --- | --- | --- | --- | --- | --- | --- | --- | --- | --- | --- |
| **Q2** | **Living arrangements** | | Living with both biological parents | | | | | |  | | Living alone with children | | | | | | |  |
| Living with one biological parent | | | |  | Single not living with children | | | | |  | | | | Living with relatives | | | |  |
| Married and living with husband and children | | | |  | Married living with children but not living with husband | | | | | | |  | | | Single living with partner and children | |  | |

| **Q3** | **Level of Education** | | No formal Education |  | Grade 1-7 | |  | Grade 8-12 |  |
| --- | --- | --- | --- | --- | --- | --- | --- | --- | --- |
| Diploma degree | |  | Undergraduate degree |  | | Post graduate degree | | |  |

| **Q4** | **Are you employed in a salary paying job?** | Yes |  | | No | |  | | |
| --- | --- | --- | --- | --- | --- | --- | --- | --- | --- |
| **Q5** | **If yes, how much do you earn per month?** |  | | | | | | | |
| **Q6** | **If no, do you have your personal business?** | Yes |  | | No | |  | | |
| **Q7** | **If yes, how much do you earn in a month?** |  | | | | | | | |
| **Q8** | **Do you receive any government social grant?** | Yes |  | | No | |  | | |
| **Q9** | **If no, do you receive monetary support from friends or family member?** | | | Yes | |  | | No |  |
| **Q10** | Which of the following best describes your **main work** status over the past 12 months? | Government employee (1)  Non-government employee (2)  Self-employed (3)  Student (4)  Retired (5)  Unemployed (6)  Others Specify ………………….. | | | | | | | |

**Measures of social capital**

| **Q11** | Please indicate the extent to which you agree to the following statements | SA | A | N | D | SD |
| --- | --- | --- | --- | --- | --- | --- |
| a. | I meet socially with friends, relatives or work colleagues at least once a week |  |  |  |  |  |
| b. | I have at least one close friend |  |  |  |  |  |
| c. | I regularly stop and talk with people in neighbourhood |  |  |  |  |  |
| d. | I belong to a social network group |  |  |  |  |  |
| e. | I am satisfied with my family life |  |  |  |  |  |
| f. | I am satisfied with my social life |  |  |  |  |  |
| g. | I have a spouse, family member or friend to rely on if I have a serious problem |  |  |  |  |  |
| h. | I give special help to at least one sick, disabled or elderly person living or not living with them |  |  |  |  |  |
| i. | I borrow things and exchange favours with my neighbours |  |  |  |  |  |

Measure of social wellbeing

| **Q12** | Please indicate the extent to which you agree to the following statements | SA | A | N | D | SD |
| --- | --- | --- | --- | --- | --- | --- |
| a. | I feel happy |  |  |  |  |  |
| b. | I have a lot of fun |  |  |  |  |  |
| c. | I love life |  |  |  |  |  |
| d. | I am a cheerful person |  |  |  |  |  |
| e. | I feel upset about things |  |  |  |  |  |
| f. | I feel I do things wrong a lot |  |  |  |  |  |
| g. | I feel unhappy a lot of the time |  |  |  |  |  |
| h. | I worry a lot that other people might not like me |  |  |  |  |  |
| i. | I worry about being stigmatised |  |  |  |  |  |
| j. | I worry about what other people might be saying about me |  |  |  |  |  |
| k. | I worry a lot about things at home |  |  |  |  |  |
| l. | I worry a lot about mistakes that I make |  |  |  |  |  |
| m. | I keep my feelings to myself |  |  |  |  |  |
| n. | When I am feeling happy, I am careful not to show it |  |  |  |  |  |
| o. | I control my feelings by not showing them |  |  |  |  |  |
| p. | When I’m feeling bad (e.g sad, angry, worried), I’m careful not to show it |  |  |  |  |  |
| q. | In most ways my life is close to the way I would want it to be |  |  |  |  |  |
| r. | The things in my life are excellent |  |  |  |  |  |
| s. | I am happy with my life |  |  |  |  |  |
| t. | So, far I have gotten the important things I want in life |  |  |  |  |  |
| u. | If I could live my life over, I would have it the same way |  |  |  |  |  |
| v. | I have more good times than times |  |  |  |  |  |
| w | I believe more good things than bad thing will happen to me |  |  |  |  |  |
| x | I believe that things will work out, no matter how difficult they seem |  |  |  |  |  |
| y | I finish whatever I start |  |  |  |  |  |
| Z | I keep at my work until I’m done with it |  |  |  |  |  |
| ba. | Once I make a plan to get something done, I stick to it |  |  |  |  |  |
| bb. | I am a hard worker |  |  |  |  |  |

Section B: To be completed by mothers

| Q13 | Year diagnosed with HIV |  | Year first initiation of HAART | |  |
| --- | --- | --- | --- | --- | --- |
| Q14 | Previous regimen |  | Current regimen |  | |

Adherence to medications

| Q15 | Since delivery, do you sometimes forget to use your ARV medication? | | Yes |  | No |  |
| --- | --- | --- | --- | --- | --- | --- |
| Q16 | Over the past two weeks, were there any days you did not take your ARV medication? | | Yes |  | No |  |
| Q17 | Have you ever cut back or stop taking your ARV medication because you felt worse when you took it? | | Yes |  | No |  |
| Q18 | When you travel or leave home, do you sometimes forget to bring along your ARV medication? | | Yes |  | No |  |
| Q19 | Did you use your ARV medication yesterday? | | Yes |  | No |  |
| Q20 | When you feel healthy, do you sometimes stop taking your ARV medication? | | Yes |  | No |  |
| Q21 | Do you often have difficulty remembering to take all your ARV medication? | Yes | |  | No |  |

| Q22a | PP viral load 1 |  | PP viral load 2 |  | | PP viral load 3 |  |
| --- | --- | --- | --- | --- | --- | --- | --- |
| Q22b | PP CD4 count |  | PP CD4 count 2 |  | | PP CD4 count 3 |  |
| Q22c | Hb |  | Weight |  | | Height |  |
| Q22d | Arm Span |  | Neck Circumference` | |  |  |  |

| Q23 | Do you have knowledge of viral load? | Yes |  | No |  |
| --- | --- | --- | --- | --- | --- |
| Q24 | Do you think your viral load is suppressed? | Yes |  | No |  |

| Q25 | Have you been hospitalised since you delivered your baby? | | Yes |  | No |  |
| --- | --- | --- | --- | --- | --- | --- |
| Q26 | If yes, how many times have you been hospitalised? | |  | | | |
| Q27 | What were the reasons why you were hospitalised? |  | | | | |
| Q28 | What treatment did you receive? |  | | | | |

| Life Style Assessment Questions | | | | | | | | |
| --- | --- | --- | --- | --- | --- | --- | --- | --- |
|  | **Questions** | **Responses** | | | | | | |
| Q29 | Have you ever smoked? | Yes |  | | No | |  | |
| Q30 | Do you currently smoke any tobacco product such cigarette, cigar, Dagga? | Yes |  | | No | |  | |
| Q31 | How old were you when you first started smoking? |  | | | | | | |
| Q32 | Do you currently smoke daily? | Yes |  | | No | |  | |
| Q33 | On average, **how many** of the following do you smoke each day? | Manufactured cigarettes **└─┴─┘** | | | | | | |
|  |  | Hand-rolled cigarettes **└─┴─┘** | | | | | | |
|  |  | Pipes full of tobacco  **└─┴─┘** | | | | | | |
|  |  | Dagga **└─┴─┘** | | | | | | |
| Q34 | In the past, did you **ever** smoke **daily**? | Yes |  | | No | |  | |
| Q35 | During the past 7 days, on how many days did someone **in your home** smoke when you were present? | Number of days  **└─┴─┘** | | | | | | |
| Q36 | During the past 7 days, on how many days did someone smoke in closed areas **in your workplace** (in the building, in a work area or a specific office) when you were present? | Number of days  **└─┴─┘**  Don't know or don't work in a closed area 77 | | | | | | |
| Alcohol Consumption | | | | | | | | |
| Q37 | Have you **ever** consumed an alcoholic drink such as beer, wine, spirits, fermented cider. | Yes | |  | | No | |  |
| Q38 | Have you consumed an alcoholic drink within the **past 12 months**? |  | | | | | | |
| Q39 | During the past 12 months, **how frequently** have you had at least one alcoholic drink? | Daily  5-6 days per week  1-4 days per week  1-3 days per month  Less than once a month | | | | | | |
| Q40 | Have you consumed an alcoholic drink within the **past 30 days**? | Yes | |  | | No | |  |
| Q41 | During the past 30 days, how many times did you have  for **men**: **five or more**  for **women**: **four or more**  standard alcoholic drinks in a single drinking occasion? | Number of times └─┴┘ | | | | | | |

| **Fertility intention, pregnancies and abortions** | | | | | | | | | | |
| --- | --- | --- | --- | --- | --- | --- | --- | --- | --- | --- |
| **Q42** | Do you desire to have more children? | Yes | |  | | | No | |  | |
| **Q43** | If yes, when do you intend to have more children | Now |  | | Later |  | | Unsure | |  |
| **Q44** | Have you become pregnant since the delivery of your child? |  |  | |  |  | |  | |  |
| **Q45** | Was the pregnancy intended? |  |  | |  |  | |  | |  |
| **Q46** | What did you do to the pregnancy? | Keep it | | |  | Abort it | | | |  |

| Contraceptive use | | | | | | | |
| --- | --- | --- | --- | --- | --- | --- | --- |
| **Q 47** | Are you currently using any contraceptive method? | Yes |  | No | |  | |
| **Q48** | If no, why are you not using any? | \| Reasons \| Yes \| No \| \| --- \| --- \| --- \| \| Side effects \|  \|  \| \| Infrequent sex \|  \|  \| \| Not affordable \|  \|  \| \| Lack of knowledge \|  \|  \| \| Lack of interest \|  \|  \| \| Accessibility \|  \|  \| | | | | | |
| **Q49** | Which method are you currently using? | Injectable | | Yes |  | No |  |
|  |  | Implants | |  |  |  |  |
|  |  | Oral Pills | |  |  |  |  |
|  |  | IUCD | |  |  |  |  |
|  |  | Condoms | |  |  |  |  |
|  |  | Withdraw method | |  |  |  |  |
|  |  | Rhythm method | |  |  |  |  |

| **Disclosure** | | |
| --- | --- | --- |
| **Q50** | Have you disclosed your HIV status to your partner? |  |
| **Q51** | Have you disclosed your HIV status to your family members? |  |
| **Q52** | Have you disclosed your HIV status to your religious leaders? |  |
| **Q53** | Have you disclosed your HIV status to those that you live with? |  |
| **Q54** | If you answered “No” in Q50, are there reasons why you have not disclosed yet? Document the responses of the participants. | |

Section 2: Infant assessment

| Q54 | Age of baby in months | | | | |  | | Weight (Kg) | | | | | | | | |  | | | | | Length | | | | |  | | Head (cm) | | | | | | |  |  |
| --- | --- | --- | --- | --- | --- | --- | --- | --- | --- | --- | --- | --- | --- | --- | --- | --- | --- | --- | --- | --- | --- | --- | --- | --- | --- | --- | --- | --- | --- | --- | --- | --- | --- | --- | --- | --- | --- |
|  | Gender | | Male | | |  | | Female | | | | |  | | | | | Received immunisation | | | | | | | | | | Yes | | |  | | No | |  | |  |
| Q55 | Birth PCR done | Yes | |  | | | No | | | |  | | | | | Birth PCR result | | | | | | | | | Positive | | | |  | | Negative | | | | |  |  |
| Q56 | 12 weeks PCR done | | | | Yes | |  | | | No | | | | |  | | 12 PCR result | | | | | | | | | Positive | | |  | | Negative | | | | |  |  |
| Q57 | Prophylaxis given to baby | | | | | | Yes | |  | | No | | | | | |  | | |  | | | | | | | | | |  | |  | | | |  | |
| Q58 | Name of prophylaxis | | | | | | Nevirapine | | | | | | |  | | | AZT | | | | | | Nevirapine &AZT | | | | | |  | | | | | | | | |
| Q59 | Duration of prophylaxis (in months) | | | | | | | | | | |  | | | | | | | | | | | | | | | | | | | | | | | | | |
| Q60 | Duration of exclusive breast feeding? | | | | | | | | | | | | | | | | | |  | | | | | | | | | | | | | | | | | | |
| Q61 | Duration of exclusive formula feeding | | | | | | | | | | | | | | | | | |  | | | | | | | | | | | | | | | | | | |
| Q62 | When did you introduce formula feeding? | | | | | | | | | | | | | | | | | |  | | | | | | | | | | | | | | | | | | |
| Q63 | When did you start giving the baby solid | | | | | | | | | | | | | | | | | |  | | | | | | | | | | | | | | | | | | |
| Q64 | What were the main reasons you introduce formula feeding at the time you started giving the baby formula? | | | | | | | | | | | | | | | | | |  | | | | | | | | | | | | | | | | | | |
| Q65 | Has the baby been hospitalised? | | | | | | | | Yes | | | | | |  | | | | No | |  | | | Number of times baby has been hospitalised? | | | | | | | | | |  | | | |
| Q66 | Reason for hospitalisation? | | | | | | | | | | | | | | | | | |  | | | | | | | | | | | | | | | | | | |
| Q67 | Assessment of cognitive development | | | | | | | | | | | | | | | | | |  | | | | | | | | | | | | | | | | | | |
| Q68 | Assessment of motor development | | | | | | | | | | | | | | | | | |  | | | | | | | | | | | | | | | | | | |
| Q69 | Assessment of language development | | | | | | | | | | | | | | | | | |  | | | | | | | | | | | | | | | | | | |
